# The Role of Fat-Soluble Vitamins in the Prevention and Management of Atopic Dermatitis

**DOI:** 10.3390/ijms27188399

**Published:** 2026-09-21

**Authors:** Caitlyn Cardenas, Alexandria K. Vo, Sarah Siddiqui, Kota V. Ramana

**Affiliations:** Department of Biomedical Sciences, Noorda College of Osteopathic Medicine, Provo, UT 84606, USA

**Keywords:** atopic dermatitis, eczema, fat-soluble vitamins, skin, inflammation, oxidative stress

## Abstract

Atopic dermatitis (AD) is a chronic inflammatory skin disorder characterized by epidermal barrier dysfunction, immune dysregulation, and increased oxidative stress. The current treatment strategies include topical corticosteroids, calcineurin inhibitors, and systemic immunomodulators. However, long-term use of steroids and other immunomodulators has unwanted side effects, and therefore recent interest has shifted towards complementary natural therapeutic approaches. Several studies indicate that natural plant-based antioxidants may help prevent AD. Specifically, a few studies demonstrated the significance of fat-soluble vitamins (A, D, E, and K) in controlling AD, owing to their strong roles in antioxidant defense, immune regulation, and maintenance of epidermal barrier integrity. In this review, we discussed recent evidence demonstrating the significant biological and therapeutic roles of fat-soluble vitamins in AD. We also discussed how vitamins A, D, E, and K may influence multiple mechanisms involved in AD pathogenesis and their potential as adjuvant therapy. Based on limited preclinical and clinical data, additional well-designed mechanistic and clinical trials are needed to establish their therapeutic efficacy in treating AD.

## 1. Introduction

Atopic dermatitis (AD) is a chronic, relapsing inflammatory disorder characterized by pruritus, dryness, and eczematous lesions [1]. Disruption of the epidermal barrier and dysregulated immune and oxidative responses are major causes of AD progression [2,3]. AD is now recognized as one of the most common dermatologic conditions worldwide, which is frequently associated with food and environmental allergies [4]. It can also be due to personal or family history of atopy [5]. Generally, AD may begin in early childhood and could persist into adulthood, with an estimated prevalence of ~20% in children and 1–3% in adults [6,7]. Although AD affects cutaneous manifestations, it can substantially affect sleep and psychosocial well-being and is associated with an increased risk of other allergic diseases, including asthma and allergic rhinitis [8,9].

**Figure 1:**
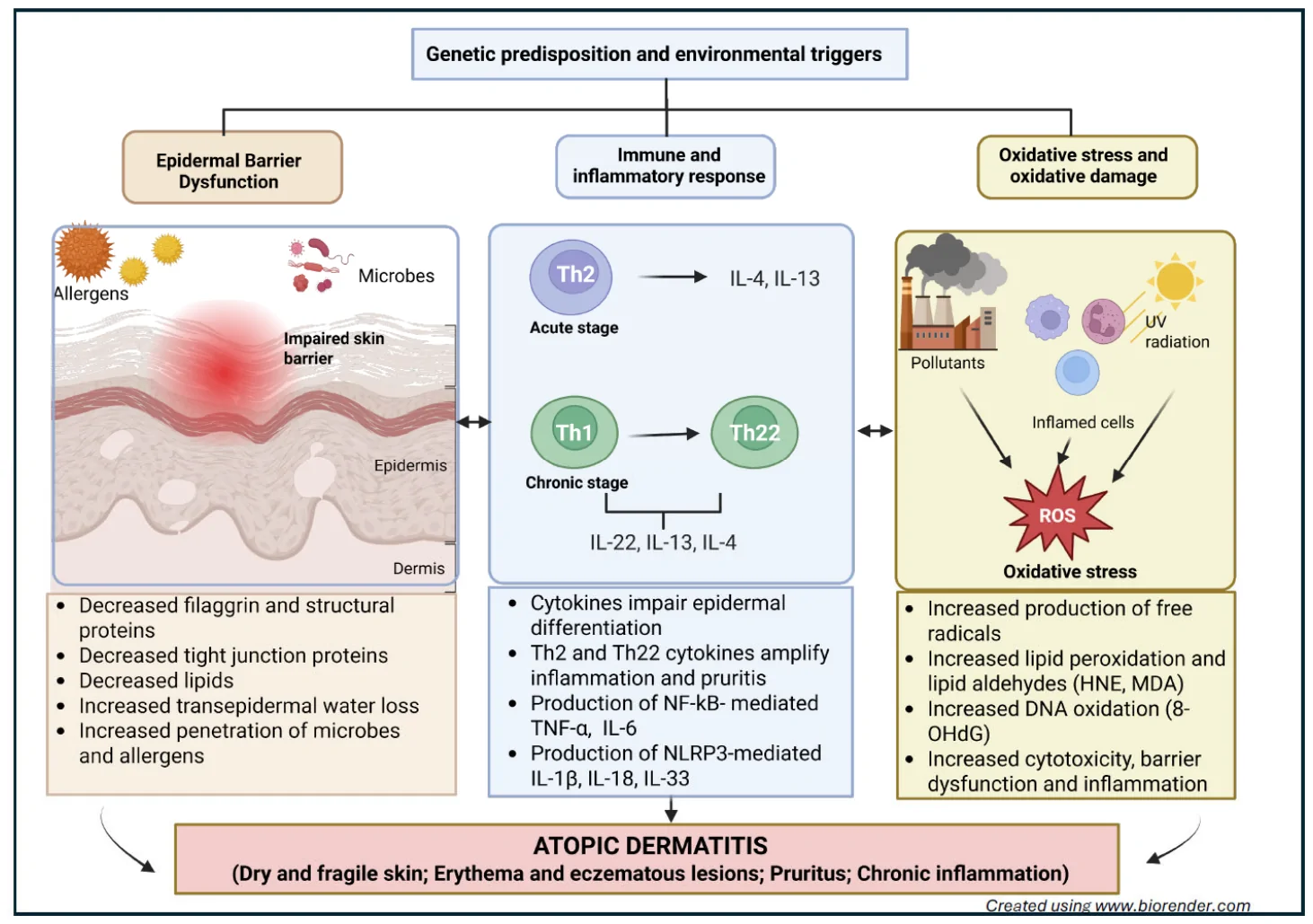
**Pathogenesis of Atopic Dermatitis.** Atopic dermatitis (AD) results from epidermal barrier dysfunction, immune dysregulation, and oxidative stress. Impaired epidermal barrier function could increase trans-epidermal water loss and facilitate penetration of allergens and microbes. Further, immune dysregulation promotes inflammatory cytokine production, mast cell activation, and pruritus. Increased reactive oxygen species (ROS) contribute to cellular damage and impairment of epidermal barrier function. These processes lead to persistent inflammation, dry and fragile skin, eczematous lesions, and intense pruritus in AD. This figure was created using www.Biorender.com (accessed on 27 August 2026).

Several studies indicate that the pathophysiology of AD involves interactions among epidermal barrier dysfunction, immune dysregulation, genetic predisposition, and environmental triggers that cause oxidative stress (Figure 1) [2,10,11]. Epidermal barrier defects are one of the major causes of AD [12]. Epidermal barrier dysfunction could be associated with filaggrin mutations, reduced ceramide levels, and increased trans-epidermal water loss. These events have been shown to facilitate the invasion of allergens and microorganisms into the skin, leading to disease pathology [13]. Further, immune cell dysregulation, particularly involving T helper 2 (Th2)-mediated responses, has been shown to cause inflammation and pruritus [14]. Moreover, environmental pollutants, extreme climates, irritants, and allergens could disrupt redox status and cause oxidative stress, which may also influence disease development and severity [14,15,16].

Current treatments for AD include topical corticosteroids and calcineurin inhibitors, phototherapy, immunomodulators, and Janus kinase (JAK) inhibitors [17,18]. However, their long-term use could be impacted by some adverse effects [19,20,21]. Therefore, recent studies have focused on understanding complementary adjunct therapeutic approaches that regulate molecular pathways involved in AD pathogenesis.

Recent studies indicate that oxidative stress is one of the potential contributors to the development and progression of AD [22,23,24]. Indeed, several plant-derived natural antioxidants have been shown to prevent AD by modulating the oxidative and inflammatory responses implicated in the disease pathology [25,26,27]. Some antioxidants have been shown to improve epidermal barrier function [28]. Recent studies also indicate the importance of fat-soluble vitamins A, D, E, and K in preventing complications associated with oxidative stress and inflammation, including inflammatory diseases and cancers [29,30]. Because of their strong antioxidant and immunomodulatory activities, these vitamins could play a significant role in controlling various skin disorders, including AD. Indeed, some studies indicate vitamin A and D deficiency in patients with AD [31,32,33]. Ye et al. [34] showed the significance of maternal antioxidant vitamin intake and infant AD. They showed a significant linear relationship between higher first-trimester vitamin E concentrations and reduced risk of infant AD.

In this review, we discussed recent studies on the roles of fat-soluble vitamins A, D, E, and K in AD. We place special emphasis on their effects on oxidative stress, immune regulation, epidermal barrier function, and their potential use as adjunctive therapeutic strategies. We conducted a PubMed search to identify relevant studies published within the last 10 years, with a few exceptions from 2000 to 2015 that were included because of their important foundational, mechanistic, or clinical relevance. Embase and Scopus were not systematically searched, as the objective of this review was to provide a focused narrative synthesis of the current biomedical literature rather than an exhaustive systematic review. Predefined keywords included “atopic dermatitis,” “eczema,” “antioxidants,” “vitamin A,” “vitamin D,” “vitamin E,” and “vitamin K.” Only original preclinical research articles, randomized and nonrandomized clinical studies, reviews, systematic reviews, and meta-analyses were included. We excluded studies that involve water-soluble vitamins and those on inflammatory skin conditions other than AD.

## 2. Pathogenesis of Atopic Dermatitis

### 2.1. Epidermal Barrier Dysfunction

The epidermal barrier is important for maintaining cutaneous homeostasis and preventing excessive water loss [35]. It also hinders the penetration of environmental allergens and pathogens. Thus, it acts as the first line of defense against pathogens, pollutants, and allergens. Epithelial barrier dysfunction is more common in patients with AD; as a result, patients are highly sensitive to environmental exposures and microbial pathogens Figure 1; [36,37]. Thus, epithelial barrier damage leads to skin dryness and desquamation because of transdermal water loss. This increases pollutant and pathogen penetration, leading to significant inflammation. Further, mutations in the filaggrin (FLG) gene, which helps maintain the epidermal permeability barrier, have been shown to cause barrier dysfunction [38,39]. Filaggrin contributes to keratin aggregation, cornified-envelope formation, and natural moisturizing factor (NMF) generation, which maintains stratum corneum hydration and an acidic skin surface. Some of the most common *FLG* loss-of-function (LOF) variants include R501X and 2282del4, specifically in individuals of European ancestry, whereas variants such as R2447X, S3247X, and 3702delG have been reported with differing frequencies across populations [40,41,42,43]. Functionally, *FLG* deficiency could reduce NMF, impair stratum corneum hydration and acidification, and increase trans-epidermal water loss and penetration of environmental allergens, irritants, and microorganisms, accordingly promoting immune sensitization and cutaneous inflammation [44,45].

Similarly, decreased ceramide levels could also contribute to impaired barrier integrity [46]. In addition, impaired cell-to-cell contact junctions due to the absence or dysfunction of claudins and cadherins could also increase barrier dysfunction [47]. Claudin-1 (CLDN1), a major component of epidermal tight junctions, is functionally impaired in AD skin, increasing paracellular permeability and helping penetration of allergens and microbial products. Type 2 cytokines interleukin (IL)-4 and IL-13 can further decrease tight-junction proteins, including CLDN1, CLDN4, and occludin (OCLN), partly through JAK–STAT6 signaling, linking inflammation directly to worsening barrier dysfunction [48,49]. Alterations in cadherin-mediated adhesion similarly contribute to epidermal instability. E-cadherin (CDH1) maintains keratinocyte–keratinocyte adhesion, and its disruption or proteolytic cleavage can weaken epidermal cohesion; activation of p38 MAPK–MMP-2 signaling, for example, promotes E-cadherin cleavage and loss of keratinocyte adhesion [50]. In addition, Totsuka et al. [51] showed that the reduced expression of the desmosomal cadherins desmoglein-1 (DSG1) and desmocollin-1 (DSC1) regulates epidermal cell adhesion by altering IL-4 and IL-13. Thus, *FLG* deficiency and abnormalities in tight- and adherens-junction proteins could affect barrier disruption and cutaneous inflammation in AD. Further, these processes could facilitate allergen and microbial penetration and amplify cutaneous inflammatory responses. Several studies suggest that fat-soluble vitamins play an important role in this process because they support epidermal differentiation and maintain skin integrity [52,53]. For example, vitamin A has been shown to regulate keratinocyte and epidermal proliferation and differentiation [54]. Similarly, vitamin D influences keratinocyte differentiation, tight-junction protein expression, and antimicrobial peptide production [55,56].

### 2.2. Immune Dysregulation and Inflammation

Immune dysregulation is another major contributor to AD pathophysiology [57,58]. Th1 and Th2-associated cytokines contribute to inflammation, pruritus, and disruption of normal epidermal function. During acute phases of AD, Th2 cytokines such as IL-4 and IL-13 predominate, whereas in chronic stages Th1 responses are limited while Th2 and Th22 cytokines are significantly elevated [59]. These cytokines could impair epidermal differentiation. Some studies also indicate that increased cytokines such as IL-22 in the serum of AD patients contribute to skin inflammation [60,61]. Further, activation of nuclear factor kappa B (NF-κB) and toll-like receptor 4 (TLR4) signaling has been shown to be associated with AD pathology [62,63]. Similarly, increased NLR family pyrin domain containing 3 (NLRP3)-inflammasome-mediated innate immune cytokines have been associated with the inflammatory response in AD [64,65]. These immune abnormalities provide a potential mechanistic basis for investigating nutrients that can modify inflammatory and immune responses. For example, vitamins D and K have been shown to be particularly important for immune cell modulation and inflammatory response [66,67,68,69]. Indeed, several studies have shown that vitamin D receptors expressed in keratinocytes and immune cells can influence gene transcription and immune regulation [70,71]. Similarly, vitamin A has also been reported to influence lymphocyte proliferation and cytokine production [72], suggesting a possible role in regulating the inflammatory response in AD.

### 2.3. Oxidative Stress in Atopic Dermatitis

Skin is constantly exposed to environmental oxidants and pollutants that significantly alter the balance between reactive oxygen species (ROS) production and endogenous antioxidant defenses, leading to oxidative stress. Several studies have shown that increased oxidative stress could contribute to dermal cell injury, dysfunction, and inflammation in AD [73,74]. Indeed, Li Pomi et al. [75] have shown that increased oxidative stress can promote lipid peroxidation and alter cellular signaling pathways, impairing epidermal barrier integrity. Several studies indicate that lipid peroxidation-derived lipid aldehydes such as hydroxynonenal and malondialdehyde, as biomarkers of oxidative stress, and 8-hydroxydeoxyguanosine (8-OHdG) as a biomarker for DNA oxidation were elevated in patients with AD and other skin diseases [76,77,78,79]. These studies suggest that oxidative stress has a crucial role in AD and indicate the potential role of antioxidants and vitamins in controlling AD pathogenesis. For example, Vitamin E is a potent lipid-soluble antioxidant that neutralizes lipid-peroxidizing radicals and protects cellular membranes from oxidative damage [80]. Similarly, carotenoids, which are vitamin A precursors, may also contribute to ROS scavenging [81]. In addition, vitamin D has been shown to participate in cellular redox processes that could restore oxidative stress-mediated barrier dysfunction and inflammation in AD [82].

## 3. Current Management of Atopic Dermatitis

Currently, emollients and moisturizers remain a major part of AD management, as they help restore epidermal barrier function and reduce flare frequency [83,84]. Further, identifying and preventing individual triggers are also important components of disease management and prevention.

Several studies indicate that topical corticosteroids are commonly used as first-line therapy in AD [85,86]. Corticosteroids such as clobetasol have been shown to treat acute flares and reduce inflammation, itching, and erythema by suppressing inflammatory responses [87]. Frequently prescribed agents include hydrocortisone [88], triamcinolone [89], and betamethasone [90]. However, prolonged or inappropriate corticosteroid use may cause adverse cutaneous effects, including skin atrophy [91,92].

Further, topical calcineurin inhibitors, including tacrolimus and pimecrolimus, provide an alternative, particularly for sensitive areas such as the face and intertriginous regions [93]. Some studies indicate that calcineurin inhibitors have a lower risk of skin atrophy than topical corticosteroids [94]. Recent studies also suggest that phototherapy, particularly using narrowband ultraviolet B radiation (UVB), could be therapeutic for moderate-to-severe AD disease that is refractory to topical therapy [95,96]. However, phototherapy has some limitations, including accessibility, time commitment, and possible long-term risks related to photoaging and carcinogenesis [97]. In addition, systemic immunosuppressive agents, such as cyclosporin and methotrexate, may be effective in severe AD [98,99]. However, they could also be associated with potentially significant adverse effects. Recent biologic therapies such as dupilumab target IL-4/IL-13 signaling and have substantially expanded treatment options for moderate-to-severe AD [100]. JAK pathway inhibitors have also been shown to be another therapeutic approach for AD [101].

Despite recent therapeutic advances, their use has limitations due to adverse effects and lifestyle burden. Therefore, novel adjunctive therapeutic approaches using natural antioxidants are needed to avoid unnecessary side effects of corticosteroids and immunosuppressants. Recent studies have shown that fat-soluble vitamins are of particular interest because of their potential effects on antioxidant defense, inflammatory signaling, immune regulation, and epidermal barrier function.

## 4. Fat-Soluble Vitamins in Atopic Dermatitis

### 4.1. Vitamin A

Vitamin A is a fat-soluble vitamin that exists in several biologically active forms such as retinol, retinal, and all-trans-retinoic acid [102]. These compounds contribute to vision, reproduction, immune function, and epithelial homeostasis [103]. Within skin, vitamin A regulates keratinocyte proliferation and differentiation, epidermal turnover, and sebaceous gland function [104,105]. Vitamin A deficiency can appear as xerosis, impaired wound healing, and follicular hyperkeratosis [106,107]. Retinoids have been shown to promote cellular renewal and collagen production and may influence antigen-presenting capacity [108]. Carotenoids also possess antioxidant properties and may protect the skin by scavenging ROS [109].

Carotenoids could provide an additional defensive mechanism through their antioxidant activity. Carotenoids such as β-carotene, lutein, and zeaxanthin can interact with ROS and may therefore reduce oxidative damage to cellular components. This mechanism might be relevant to AD because excessive oxidative stress can promote lipid peroxidation, epidermal injury, and inflammatory signaling. Clinical observations displaying reduced circulating carotenoids and retinoids in patients with AD provide additional evidence of an association between carotenoid/vitamin A. For example, a study by Lucas et al. [110] measured plasma levels of carotenoids such as lutein and zeaxanthin and retinoids such as retinol and all-trans-retinoic acid in patients with AD. This study shows that, compared with healthy volunteers, AD patients have reduced plasma levels of carotenoids and retinoids. Similarly, Biswas et al. [111] examined skin biopsies from 86 participants, including 43 controls and 43 patients with AD. This study reports that, compared with healthy subjects, patients with AD have significantly reduced retinol levels in their skin biopsies. Additional pediatric studies have suggested associations between dietary carotenoid intake and AD. Higher beta-carotene consumption has been associated with a reduced risk of AD, and another pediatric study reported lower levels of vitamins A and D in children with AD compared with unaffected children [112,113].

Alitretinoin is a retinoid drug commercially used for patients with chronic hand eczema (CHE) resistant to topical steroidal drugs. Ferrucci et al. [114], in a multicenter clinical survey of dermatology clinics in Italy, showed that alitretinoin treats moderate-to-severe CHE refractory to potent topical corticosteroid therapy. Similarly, a prospective, multicenter, two-arm parallel-group, adaptive randomized controlled trial in UK secondary care dermatology outpatient clinics by Wittmann et al. [115] showed that alitretinoin as first-line therapy improved CHE compared with 12-week ultraviolet therapy. Further, Luchsinger et al. [116], in a retrospective analysis of patients under age 18, showed that alitretinoin is effective and safe for children with CHE refractory to topical steroid therapy. Another study by Jang et al. [117] showed that alitretinoin and cyclosporin are effective in patients with CHE. This retrospective study included patients with CHE who visited the Dermatology Department at Hanyang University Seoul Hospital in Korea between March 2013 and February 2020. Although CHE and AD are distinct conditions, their overlapping features of skin barrier dysfunction and cutaneous inflammation suggest that findings from CHE studies may provide indirect supportive evidence relevant to AD pathophysiology and treatment.

**Figure 2:**
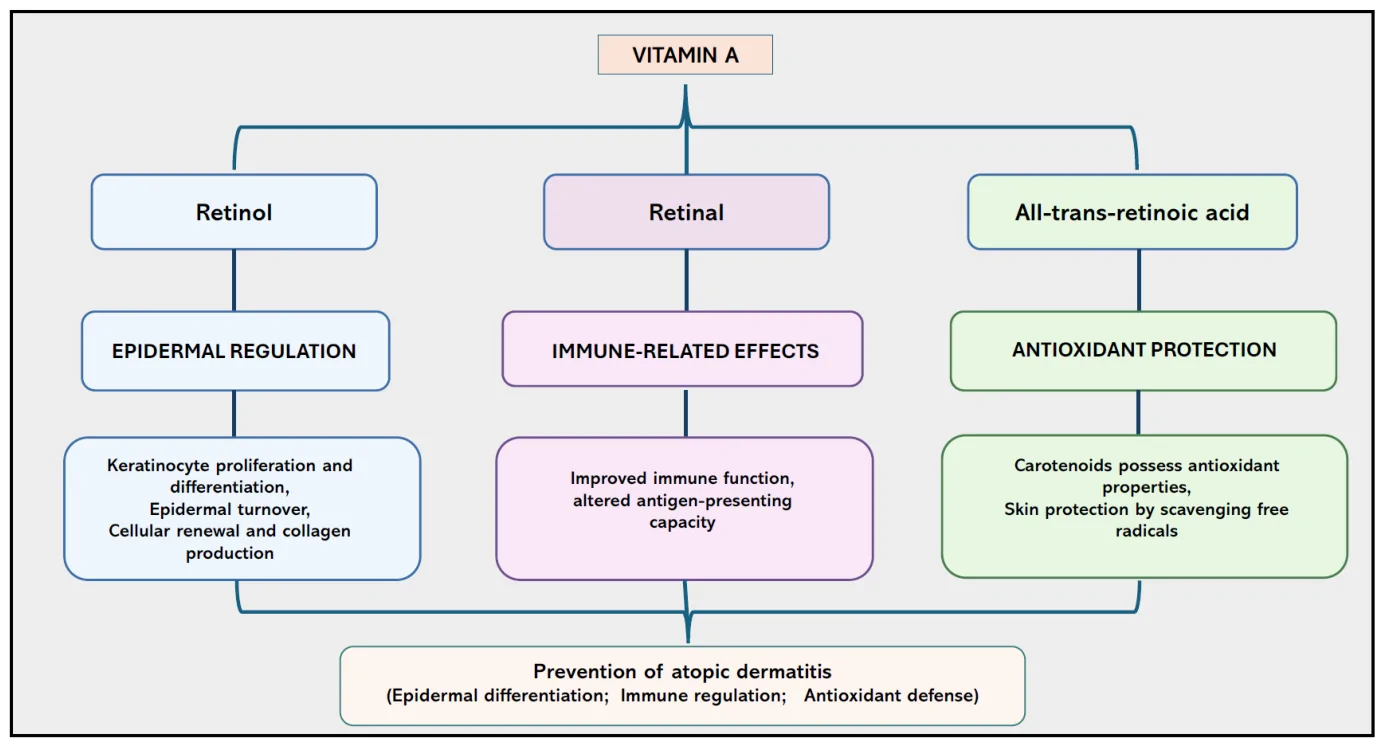
**Role of vitamin A in the prevention of atopic dermatitis.** Bioactive derivatives of vitamin A, including retinol, retinal, and all-trans-retinoic acid, contribute to skin homeostasis through multiple mechanisms. Retinol supports epidermal regulation by promoting keratinocyte proliferation and differentiation, epidermal turnover, cellular renewal, and collagen production. Retinal and related retinoids may modulate immune function and antigen-presenting capacity, therefore contributing to immune regulation. In addition, vitamin A–related carotenoids provide antioxidant protection by scavenging free radicals and limiting oxidative stress in the skin. Together, they help protect against AD by supporting epidermal differentiation, immune homeostasis, and antioxidant defense.

Another study by Nassar et al. [118], a single-arm, open-label study involving 20 AD patients, showed significant benefits of poly-L-lysine-based cosmetics with vitamins A and C. They showed that these vitamin combinations improved symptoms and quality of life in patients with AD. Recent studies thus suggest the beneficial effects of vitamin A in AD, as it could regulate epidermal differentiation, immune regulation, and antioxidant defense (Figure 2). However, current evidence primarily shows associations between vitamin A status and AD rather than establishing the significant efficacy of vitamin A supplementation as a treatment.

### 4.2. Vitamin D

**Figure 3:**
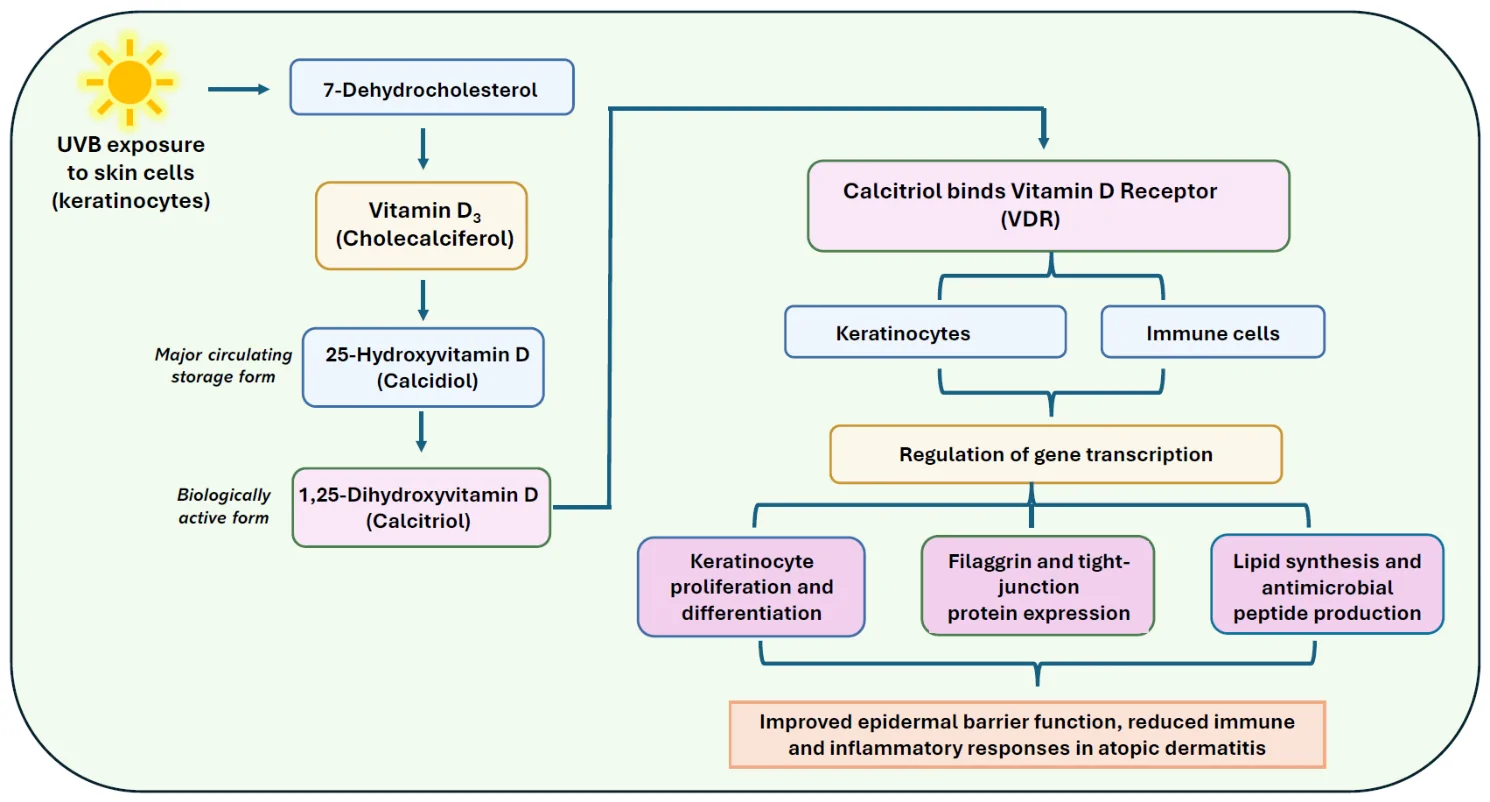
**Significance of vitamin D in epidermal barrier function and immune regulation in atopic dermatitis**. Ultraviolet B (UVB) exposure promotes the conversion of 7-dehydrocholesterol in keratinocytes to vitamin D_3_, which is metabolized to the biologically active form, 1,25-dihydroxyvitamin D (calcitriol). Calcitriol binds to the vitamin D receptor (VDR) expressed in keratinocytes and immune cells and regulates the transcription of genes involved in skin homeostasis and immune responses. Vitamin D signaling promotes keratinocyte proliferation and differentiation, enhances filaggrin and tight-junction protein expression, and antimicrobial peptide production. Thus, vitamin D could strengthen epidermal barrier integrity and could reduce dysregulated immune and inflammatory responses associated with AD.

Vitamin D is a fat-soluble vitamin and hormone that plays important roles in immune regulation and epidermal homeostasis [119]. After UVB exposure, 7-dehydrocholesterol in the skin converts to vitamin D3 [120]. Calcidiol represents the major circulating storage form, whereas calcitriol is the biologically active form [110]. Calcitriol binds to vitamin D receptors expressed in keratinocytes and immune cells and thereby regulates gene transcription [121]. Vitamin D signaling can influence keratinocyte differentiation, increase tight-junction protein expression, and stimulate antimicrobial peptide production [122,123]. These actions provide several mechanisms through which vitamin D could influence epidermal barrier function and immune responses in AD (Figure 3).

In a 2,4-dinitrochlorobenzene (DNCB)-induced mouse AD model, vitamin D metabolite, calcifediol, treatment prevents inflammatory cell infiltration and chemokine release through Signal transducer and activator of transcription 3 (STAT3) phosphorylation and AKT1/mTOR signaling. This study also indicates that calcifediol improved skin barrier function by downregulating aquaporin 3 expression [124]. Similarly, topical calcitriol application reduces dermatitis symptoms in a mouse model of NC/Nga AD [125]. Wang et al. [126] also showed that adjuvant combination therapy with vitamin D plus crisaborole significantly prevented inflammation and epidermal hyperkeratosis in a mouse model of allergic contact dermatitis compared with vitamin D- or crisaborole-alone-treated mice. Further, Trujillo-Paez et al. [127] showed that calcitriol increases the expression of tight junction proteins such as claudins in human primary keratinocytes. They also showed that calcitriol increases phosphorylation of atypical protein kinase C (PKC), Rac family small GTPase 1 (Rac1), phosphoinositide 3-kinase (PI3K), and protein kinase B (AKT), which are involved in regulating epithelial barrier dysfunction. Together, these observations support VDR, tight-junction proteins, PKC, Rac1, PI3K, and AKT as important molecular targets associated with vitamin D-mediated effects on epidermal and inflammatory processes in AD.

Several studies indicate low serum vitamin D levels are associated with AD severity [128,129,130,131,132]. A case–control study by Ahmed Mohamed et al. [133] reported an association between vitamin D deficiency and increased prevalence of AD. Maternal vitamin D intake has also been investigated in relation to the subsequent development of AD symptoms in children [134,135,136,137]. Further, low prenatal vitamin D blood levels are correlated with AD in children [138,139,140]. Blomberg et al. [138] showed that maternal prenatal 25(OH) vitamin D blood levels below 25 nmol/L are correlated with an elevated risk of AD in early childhood. They also indicated that lower vitamin D intake increases this risk into mid-childhood. Another study by Su et al. [141] showed that low serum vitamin D levels are inversely correlated with AD severity in children compared with healthy children. Wang et al. [142] showed that lower vitamin D levels in breast milk could be a risk factor for infantile AD. Similarly, Huang et al. [143] also showed that low levels of breast milk vitamin D could be a risk factor for developing AD in infants. Similarly, Jang et al. [144] found that low vitamin D levels are associated with high house dust mite sensitization in patients with severe AD. Ibrahim et al. also reported similar results [145].

Furthermore, several clinical studies have investigated whether correcting vitamin D deficiency or providing vitamin D supplementation improves AD. In a randomized double-blind study, Javanbakht et al. [146] showed that patients receiving 1600 IU/day of vitamin D for 60 days experienced significant improvement in AD symptoms compared with the placebo group. Similarly, a prospective observational study by Tsotra et al. [147] showed that vitamin D supplementation prevents symptom severity in children with AD. Another randomized, double-blind, placebo-controlled clinical trial by Sánchez-Armendáriz et al. [148] showed that patients receiving 5000 IU/day for 12 weeks, in addition to standard therapy, had strongly reduced AD symptoms. Aldaghi et al. [149], in a double-blind, randomized clinical trial, indicate therapeutic benefits of multistrain symbiotic and vitamin D supplementation in controlling AD in infants under one year. Similarly, Mansour et al. [150], in a randomized controlled trial, found that adjuvant vitamin D therapy with baseline hydrocortisone for 12 weeks significantly improved severity scores and clinical outcomes in AD. Another study by D’Auria [151] showed an association between vitamin D and AD in Italian children.

Several systematic reviews also indicate a strong association between vitamin D deficiency and AD, and support vitamin D supplementation for AD prevention. For example, Kim and Bae [152] found that vitamin D supplementation is very beneficial in reducing AD symptoms. Similarly, Kim et al. [153] found low vitamin D levels in AD patients’ serum and suggested that vitamin D supplementation could be a potential therapeutic option. Another case-controlled cross-sectional study by Sharma et al. [154] indicates an inverse relationship between serum vitamin D levels and AD index scores in patients with severe AD. A clinical study by Dogru [155] indicates that vitamin D deficiency was more common in children with moderate to severe AD and that vitamin D levels are negatively correlated with AD severity. Similarly, Hattangdi-Haridas et al. [156] found that vitamin D deficiency is associated with AD severity and that vitamin D supplementation reduced AD index scores. Ng and Yew [157] also found that lower serum vitamin D levels are associated with AD severity and that vitamin D supplementation reduces AD symptoms.

Together, the recent observations suggest a relationship between vitamin D status and AD occurrence or severity. These findings suggest a potential therapeutic role for vitamin D, although differences among studies in baseline vitamin D status, patient populations, dosing, and outcome measures should be considered when interpreting the evidence.

### 4.3. Vitamin E

**Figure 4:**
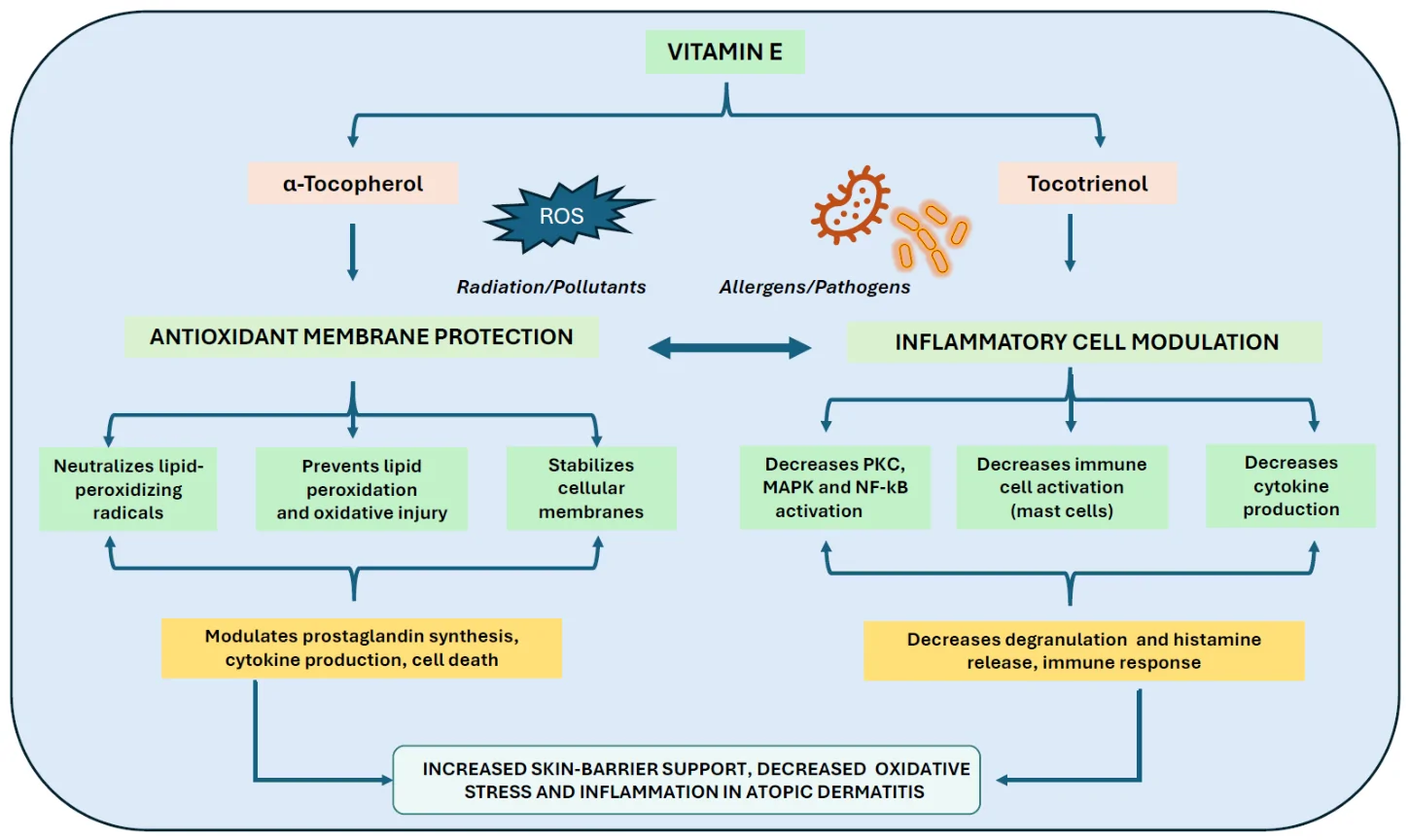
**Antioxidant and immunomodulatory roles of vitamin E in atopic dermatitis.** α-tocopherol and tocotrienols maintain skin homeostasis through antioxidant and anti-inflammatory properties. α-tocopherol protects cellular membranes by neutralizing free radicals, limiting lipid peroxidation and oxidative injury. Tocotrienols may modulate inflammatory signaling by reducing activation of PKC, MAPK, and NF-κB pathways. These actions of vitamin E could strengthen epidermal barrier function and decrease oxidative and inflammatory responses associated with AD.

Vitamin E is a lipid-soluble antioxidant that helps protect cellular membranes from oxidative damage [158,159]. Alpha-tocopherol, one of its major biologically active forms, neutralizes lipid-peroxidizing radicals and may therefore limit oxidative injury in the skin [159,160]. Vitamin E may stabilize cellular membranes, support skin-barrier function, and reduce lipid peroxidation [161]. It may modulate prostaglandin synthesis and cytokine production, providing an additional anti-inflammatory mechanism applicable to AD (Figure 4) [162].

Several preclinical studies indicate the significance of vitamin E in controlling skin inflammatory diseases in various animal models [163,164]. Hayashi et al. [165] showed that in an NC/Nga mouse model of atopic dermatitis, skin inflammation was associated with increased oxidative stress and inflammatory markers, including thiobarbituric acid reactive substances (TBARS), immunoglobulin E (IgE), cyclooxygenase (COX)-2, tumor necrosis factor-alpha (TNF-α), and NF-κB. Importantly, they showed that vitamin E levels decreased during peak disease severity. It also altered biochemical and inflammatory changes, as well as antioxidant enzymes. This study suggests that vitamin E may reduce atopic dermatitis-like inflammation by limiting oxidative stress and regulating inflammatory pathways. Thus, lipid peroxidation, COX-2, TNF-α, NF-κB, and endogenous antioxidant enzymes represent potential molecular targets associated with vitamin E activity in AD. Tocotrienols, members of the vitamin E family, may also affect allergic inflammatory pathways. For example, Tsuduki et al. [166], using an NC/Nga mouse model of allergic dermatitis, showed that tocotrienol (T3), a form of vitamin E, significantly reduced scratching, skin thickening, and serum histamine levels. Tocotrienol also suppressed mast cell degranulation and histamine release, showing a direct effect on allergic inflammatory responses. This study indicates that tocotrienol could reduce allergic dermatitis by inhibiting mast cell activation through suppression of protein kinase C (PKC) activity.

Several clinical studies have also investigated associations between vitamin E status and inflammatory skin disease [167,168]. Lower serum vitamin E levels have been reported in individuals with chronic inflammatory skin conditions, including AD. Recent studies also suggest that children and adolescents with moderate-to-severe AD may have lower vitamin E levels than those with mild disease. A cross-sectional study by Lee et al. [169] examined relationships between nutrient intake, serum vitamin E, and IgE levels in 119 Korean children aged 0–24 months with atopic dermatitis. They found that higher serum vitamin E levels were significantly associated with lower total and specific IgE levels. These data suggest that higher circulating vitamin E status may be associated with reduced allergic sensitization in children with atopic dermatitis. Another single-blind clinical study by Tsoureli-Nikita et al. [170] compared 400 IU/day of natural vitamin E with placebo for 8 months in 96 patients with atopic dermatitis. They showed that vitamin E treatment produced substantially greater clinical improvement, with 23 patients showing great improvement and 7 achieving near-complete remission. In this study, they showed that vitamin E caused a 62% reduction in serum IgE among those with the best responses. Similarly, a multicenter clinical Italian study by Nemelka et al. [171] evaluated Triderm^®^ Lenil+, a topical antioxidant skin-barrier formulation containing vitamin E, SOD, and furfuryl palmitate in 60 children aged 2 months to 14 years with AD. They have shown that Triderm^®^ Lenil+ treatment resulted in rapid and significant improvement in inflammation and eczema, including symptoms such as itching, desquamation, and xerosis. A prospective cohort study of 456 pregnant women and their infants examined the relationship between first-trimester maternal vitamin E levels and the development of atopic dermatitis during the first year of life [34]. Each 1 μg/mL increase in maternal vitamin E was associated with an 8% lower risk of infant AD. They have shown that infants of mothers in the highest vitamin E tertile had a 46% lower risk than those in the middle tertile. These results suggest that higher maternal vitamin E status during early pregnancy may protect against infant atopic dermatitis, although the observational design does not demonstrate causality.

Interestingly, some prospective studies indicate that vitamin E has not shown a significant preventive effect on AD; however, others have shown beneficial effects. For example, a prospective study by Hoppu et al. [172] examined alpha- and gamma-tocopherol levels in atopic mothers and their infants and followed the infants for atopic dermatitis and allergic sensitization during the first year of life. This study indicates that maternal vitamin E status strongly influences infant vitamin E status. However, infant tocopherol levels were not associated with atopic dermatitis or skin-prick-test responses, demonstrating no clear protective effect against atopic disease in this study. Similarly, another multicenter prospective birth cohort study by Nwaru et al. [173] examined 1133 children for serum vitamin E levels at age 1 and their association with atopy, AD, and asthma through age 6. The authors found no statistically significant associations between early vitamin E concentrations and these allergic outcomes. This study does not support a clear protective role of early-life vitamin E against atopic dermatitis and allergies. However, another prospective study by Martindale et al. [174] followed 1924 children to examine whether maternal antioxidant intake during pregnancy influenced the development of wheezing and eczema during the first two years of life. Higher maternal vitamin E intake was associated with reduced wheezing and, among children of atopic mothers, a lower risk of eczema during the second year. These conclusions suggest that maternal vitamin E intake during pregnancy may influence the risk of early childhood atopic disease. In addition, Javanbakht et al. [146] conducted a randomized, double-blind, placebo-controlled trial that evaluated vitamin D, vitamin E, and their combination for 60 days in 45 patients with atopic dermatitis. Vitamin E supplementation significantly increased erythrocyte superoxide dismutase (SOD) activity, while vitamin D increased both SOD and catalase activities; combined supplementation also improved both antioxidant enzymes. The data show that vitamin E may enhance antioxidant defense in atopic dermatitis, although vitamin D appeared equally effective and had a stronger effect on catalase activity.

Furthermore, Patrizi et al. [175] conducted a randomized, double-blind, placebo-controlled study of 44 patients with mild-to-moderate atopic dermatitis and evaluated MD2011001, a topical cream containing vitamin E, epigallocatechin gallate, and grape seed procyanidins, over 28 days. Both groups showed improvement, but MD2011001 produced a significantly faster reduction in the skin surface area affected by AD and was well tolerated. These data suggest that this vitamin E-containing antioxidant emollient may benefit mild-to-moderate AD. Similarly, Jaffary et al. [176] evaluated the beneficial effects of vitamin E (400 IU/day) for four months in 70 patients with mild-to-moderate AD in a randomized, double-blind, placebo-controlled trial. Vitamin E significantly improved itching, lesion extent, and overall SCORing Atopic Dermatitis (SCORAD) scores compared with placebo. In addition, a Mendelian randomization study by Huang et al. [177] used genome-wide association study (GWAS) data from two independent atopic dermatitis cohorts to assess whether vitamin E has a causal relationship with AD risk. Meta-analysis showed that genetically predicted higher vitamin E levels were associated with a significantly lower risk of atopic dermatitis. These findings yield genetic evidence supporting a potential protective effect of vitamin E against the development of atopic dermatitis. This study reported evidence supporting a potential causal relationship between higher vitamin E levels and lower AD risk.

Thus, clinical studies evaluated vitamin E as an adjunctive treatment for AD. Randomized controlled trials have shown that oral vitamin E supplementation was associated with reduced clinical severity and improved quality of life [146,170,176]. Other studies evaluated moisturizers containing tocotrienol-rich vitamin E in children with mild-to-moderate AD and reported improvements in disease severity and quality of life [178,179]. Thus, combined vitamin E and immunomodulatory supplementation may provide additional therapeutic effects.

### 4.4. Vitamin K

Compared with vitamins D and E, the molecular mechanisms of vitamin K in AD remain less extensively investigated. Vitamin K exists primarily as vitamin K1 (phylloquinone) and K2 (menaquinone). Beyond its established role in coagulation, vitamin K participates in the carboxylation of vitamin K-dependent proteins involved in tissue repair, vascular health, and cellular growth [180,181]. Vitamin K may also influence oxidative stress through redox cycling [182]. This antioxidant activity could protect cutaneous cells from oxidative injury and complement its anti-inflammatory effects [183]. Vitamin K is required to activate matrix Gla protein (MGP), a vitamin K-dependent protein involved in tissue and vascular homeostasis. Further, properly carboxylated MGP supports tissue stability and elasticity [184].

**Figure 5:**
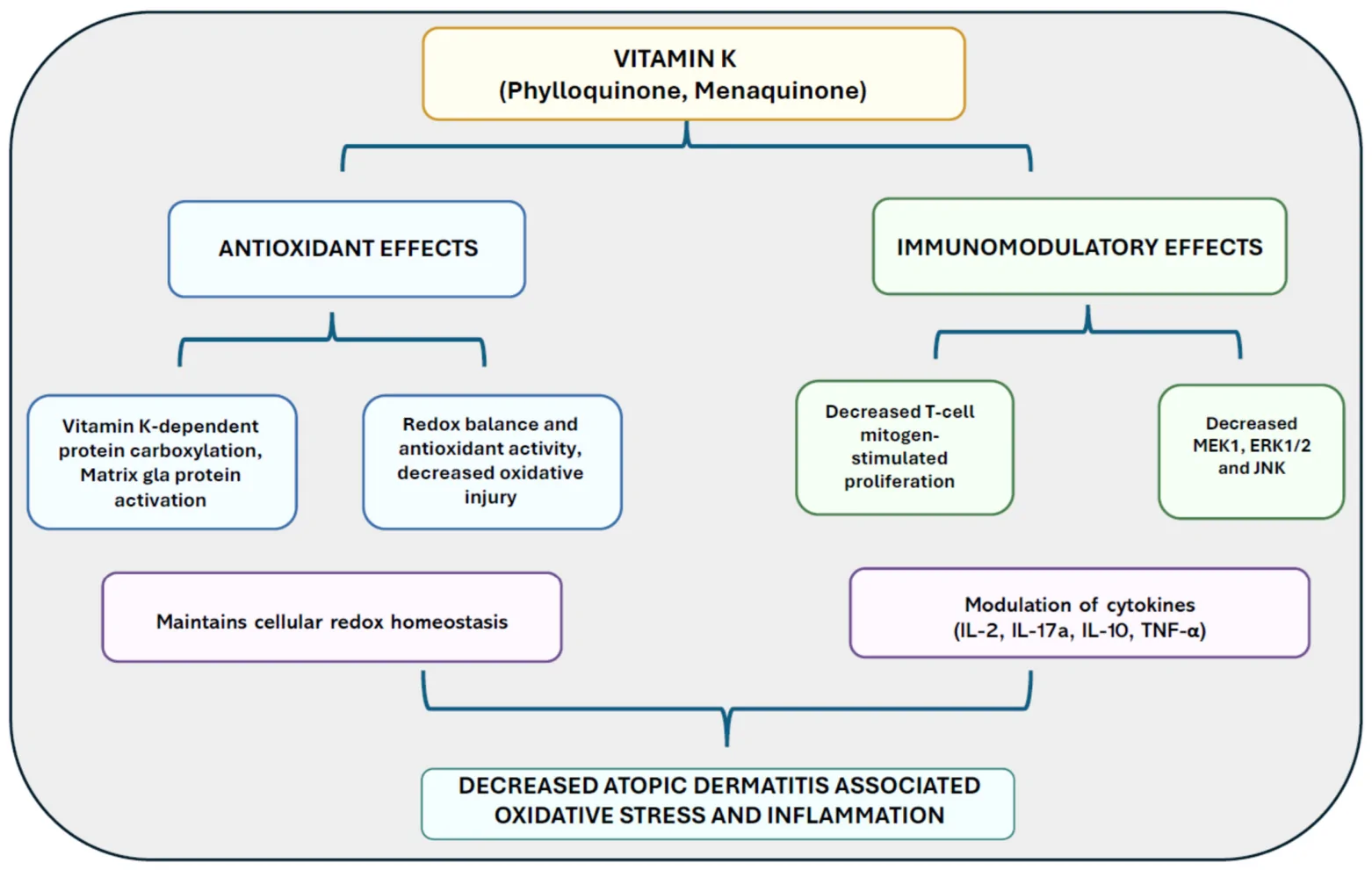
**Possible role of vitamin K in preventing atopic dermatitis.** Vitamin K can regulate antioxidant and immune-regulatory mechanisms in isolated PBMCs from AD patients. Vitamin K–dependent protein carboxylation, including activation of matrix Gla protein, together with redox-related antioxidant activity, may help maintain cellular redox homeostasis and limit oxidative injury. Vitamin K may also modulate immune responses by reducing T-cell proliferation and regulating MEK1, ERK1/2, and JNK signaling pathways. Collectively, these mechanisms suggest a role for vitamin K in reducing oxidative stress and inflammation associated with AD.

Limited evidence shows vitamin K’s role in AD prevention (Figure 5). A cell-culture study by Meng et al. [185] examined the effects of vitamin K1 and K2 on proliferation, cytokine production, and regulatory T-cell frequency in peripheral blood mononuclear cells (PBMCs) from pediatric patients with AD. The results suggest that vitamin K2 inhibited T-cell mitogen-stimulated proliferation in a dose-dependent manner, while vitamin K1 had a partial effect. Vitamin K2 also significantly reduced IL-2 production and the percentage of CD4+ and CD4+CD25+ cells in PBMCs. These results suggest that vitamin K2 regulates T-cell function in AD. Similarly, Xu et al. [186] examined the immunosuppressive effects of vitamin K2 in isolated PBMCs from AD patients. In this study, the authors report that vitamin K2 inhibits T-cell mitogen-stimulated PBMC proliferation. They similarly indicated that PBMCs isolated from AD patients showed immunosuppression to vitamin K2 compared with PBMCs isolated from healthy patients. Another study by Zhang et al. [187] suggested that vitamin K2 inhibits IL-17a, IL-10, and TNF-α and increases IL-2 in PBMCs isolated from AD patients. This study indicates that vitamin K2 decreases MEK1, (ERK)1/2, and JNK expression, suggesting that vitamin K2 prevents activated T-cell immunity in AD patients. The results identify the mitogen-activated protein kinase (MAPK) pathway, particularly MEK/ERK and JNK signaling, as a potential molecular target of vitamin K2 in AD-associated immune responses.

Thus, recent evidence suggests that vitamin K could possess immunomodulatory properties in AD. Specifically, vitamin K2 has been reported to inhibit lymphocyte proliferation and modify cytokine production in PBMCs isolated from pediatric patients with AD. These studies suggest that vitamin K may alter regulatory T-cell functions and help maintain immune homeostasis. These findings similarly suggest that vitamin K could influence AD-associated inflammation. However, additional studies are needed to investigate vitamin K’s immunomodulatory, anti-inflammatory, and potential antioxidant effects in patients with AD. Further, supporting clinical trials are needed to determine whether the mechanistic effects observed in vitro translate into meaningful clinical benefits.

Collectively, fat-soluble vitamins may influence AD through distinct biochemical mechanisms (Table 1). Together, these mechanisms converge on major components of AD pathogenesis, including oxidative stress, inflammatory signaling, immune dysregulation, and epidermal barrier dysfunction. Nevertheless, mechanistic evidence is not equally established for all fat-soluble vitamins, and clinical effectiveness should not be considered proof of a particular molecular mechanism.

**Table 1:** Biochemical Pathways and Key Molecular Targets of Fat-Soluble Vitamins in Atopic Dermatitis.

Vitamins	Biochemical Process	Key Molecular Targets	Relevance to AD	Citations
Vitamin A/retinoids	Epidermal proliferation and differentiation	Keratinocytes; retinoid-mediated cellular regulation	Maintain epidermal turnover, differentiation, and epithelial homeostasis	[104,105]
	Cutaneous immune regulation	Antigen-presenting mechanisms	Influence immune responses and epidermal homeostasis	[108]
Carotenoids	Antioxidant defense	ROS	Scavenge ROS and reduce oxidative injury to the skin	[109]
	Antioxidant/nutritional pathways	Carotenoid and retinoid status	Reduced levels are associated with AD; higher β-carotene intake reduces AD risk	[110,111,112,113]
Vitamin D	Nuclear receptor-mediated transcription	Vitamin D receptor	Regulates genes involved in keratinocyte and immune-cell function	[121]
	Epidermal barrier regulation	Claudins and other tight-junction proteins	Promotes epidermal junctional integrity and barrier function	[122,127]
	Innate antimicrobial defense	Antimicrobial peptides	Strengthens cutaneous antimicrobial defense	[123]
	Intracellular barrier signaling	atypical PKC, Rac1, PI3K, AKT	Regulates epithelial barrier-related signaling	[127]
Vitamin E	Antioxidant/lipid-peroxidation pathway	Lipid radicals, ROS	Interrupts lipid peroxidation and protects cellular membranes	[158,159,160,161]
	Inflammatory signaling	COX-2, TNF-α, NF-κB	Attenuates inflammatory responses in AD	[163,164,165]
	Endogenous antioxidant defense	Antioxidant enzymes, SOD	Supports antioxidant defenses and oxidative balance	[165]
	Mast-cell/PKC signaling	PKC, mast cells, histamine	Suppresses mast-cell degranulation and histamine release	[166]
Vitamin K	Vitamin K-dependent protein activation	Matrix Gla protein	Contributes to tissue homeostasis and stability	[184]
	Redox regulation	Oxidative/redox pathways	Protects cells against oxidative injury	[182,183]
	T-cell regulation	PBMCs, CD4+, CD4+CD25+ cells	Inhibits activated lymphocyte proliferation and modulates immune responses	[185,186]
	Cytokine regulation	IL-17A, IL-10, TNF-α, IL-2	Alters inflammatory cytokine responses in PBMCs from AD patients	[187]
	MAPK signaling	MEK1, ERK1/2, JNK	Suppresses activated T-cell signaling and inflammatory responses	[187]

## 5. Conclusions, Limitations and Future Perspectives

Recent studies suggest that fat-soluble vitamins could influence AD through several related mechanisms. Specifically, these vitamins have been shown to regulate epidermal barrier integrity and the oxidative and inflammatory responses involved in AD pathogenesis. However, the nature and mechanism of action may differ by each vitamin and its metabolic function in the body. Vitamins A, D, and E currently have stronger clinical support, whereas evidence for vitamin K remains limited, and additional studies are needed to comprehend its role in AD.

Some studies suggest that vitamins may be more beneficial when used as an adjunct to established AD therapies, such as topical corticosteroids. However, no studies strongly support replacing conventional treatment with fat-soluble vitamin supplementation. Further, well-designed clinical trials are needed to establish causal relationships with patients’ skin inflammatory type and optimal dosing strategies.

Recent literature suggests that fat-soluble vitamins may prevent AD through their antioxidant, anti-inflammatory, immunomodulatory, and skin barrier-supporting properties. For example, vitamin A and its derivatives regulate epidermal proliferation and immune responses. Vitamin D regulates skin barrier integrity and inflammatory responses. Similarly, vitamin E may protect by reducing oxidative stress and potentially attenuating inflammatory responses, whereas evidence on vitamin K in AD remains very limited. Some clinical studies generally support possible benefits of these vitamins, particularly vitamins D and E; however, findings are not entirely consistent, and many studies show associations or clinical effectiveness rather than definite mechanisms of action. Moreover, well-designed mechanistic studies and large randomized controlled trials are needed to define the molecular mechanisms involved and determine whether fat-soluble vitamins can be effective adjunctive strategies for preventing or managing AD.

Although recent studies suggest a strong correlation between decreased or deficient fat-soluble vitamin levels and AD severity in children and adults, several important limitations remain. Currently available studies are limited by patient age, disease severity, dose and duration of supplementation, and clinical scoring indices. In addition, many reported studies involve relatively small sample sizes, suggesting a need for large-scale studies. Such studies will expand the generalizability of their findings.

Furthermore, vitamin levels depend on dietary intake and the types of food consumed. Thus, most studies indicate that reduced circulating vitamin levels in patients with AD may contribute to disease pathogenesis; however, these levels may reflect dietary patterns, disease severity, oxidative stress, and systemic inflammation. Additional Mendelian randomization studies are needed to understand the causal influence of vitamins in AD pathology. Moreover, future studies should prioritize large-scale randomized controlled trials based on established clinical outcomes such as the Scoring AD Index (SCORAD) or the Eczema Area and Severity Index (EASI). Understanding the baseline vitamin status will help to determine whether therapeutic responses differ between deficient and vitamin-replete patients. Specifically, several vitamin levels decrease during pregnancy, and supplementation may help control various diseases, including inflammatory skin diseases.

Further studies are needed to investigate potential synergistic effects of combining fat-soluble vitamins alone or as an adjuvant with baseline therapies. Further, using fat-soluble vitamins alongside strong natural plant-based antioxidants such as curcumin and quercetin could improve epithelial barrier integrity and reduce oxidative and inflammatory responses leading to AD. Future studies correlating clinical outcomes with biomarkers of oxidative stress, inflammatory cytokines, epidermal barrier function, and vitamin status may provide a more complete understanding of how these nutrients influence AD pathogenesis and treatment response.

## Data Availability

No new data were created or analyzed in this study. Data sharing is not applicable to this article.
